# Supplementary material for: Mechanistic Intimate Insights into the Role of Hydrogen Sulfide in Alzheimer’s Disease: A Recent Systematic Review
Source: Int J Mol Sci. 2023 Oct 23;24(20):15481. doi: 10.3390/ijms242015481 (PMC10607039; doi:10.3390/ijms242015481)
Supplement: Supplementary file 1 [file ijms-24-15481-s001.zip › ijms-2653244-supplementary.pdf]

Mechanistic Intimate Insights into the Role of Hydrogen Sulfide in Alzheimer's Disease: A Recent Systematic Review

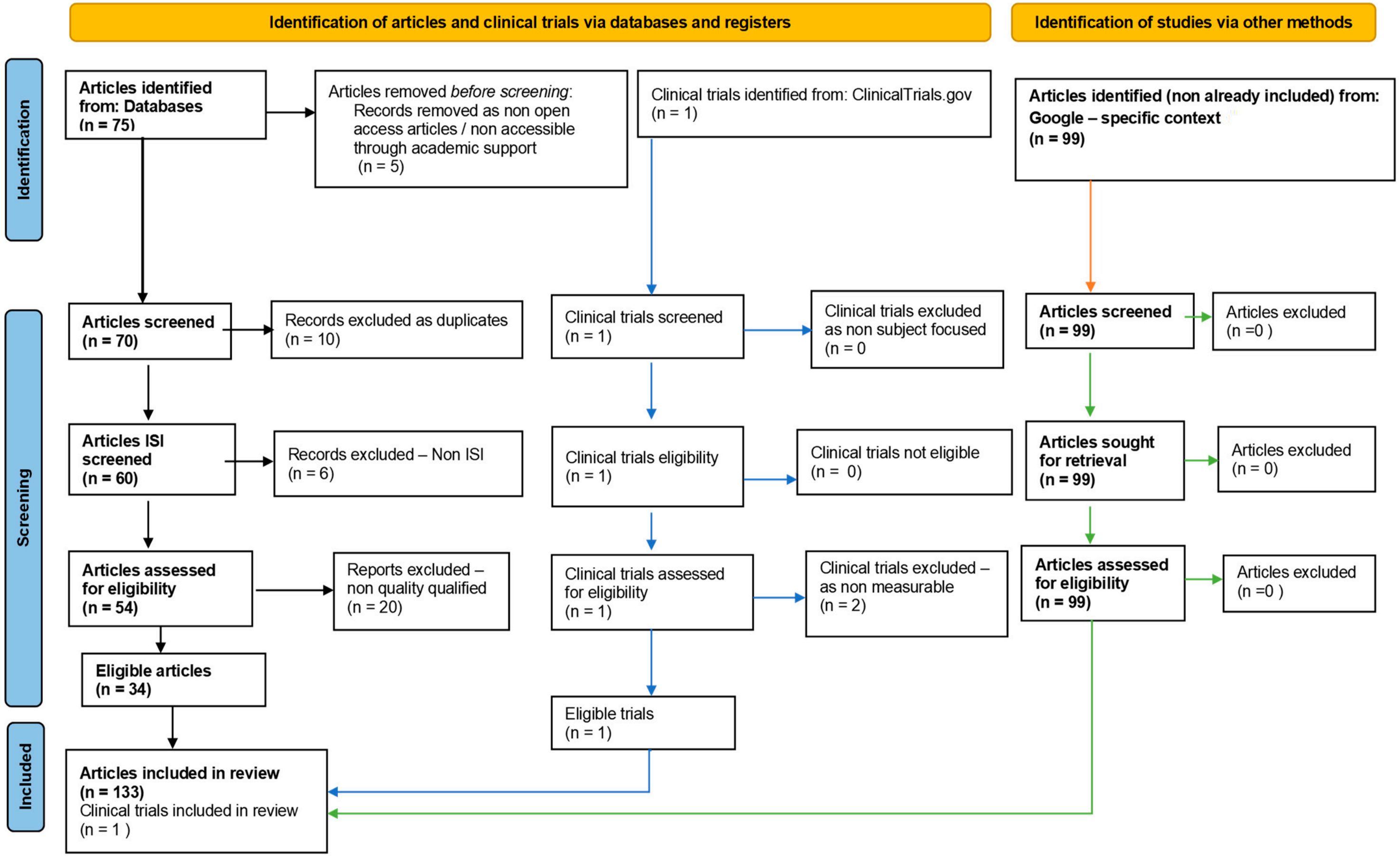

Figure S1. PRISMA data flow diagram.
